# Laser-Generated Ultrashort Pulsed Electron Beams Induce p53-Related Alteration of DNA Repair and Cell Death Pathways in Non-Small Cell Lung Cancer Cells

**DOI:** 10.3390/ijms27167215

**Published:** 2026-08-13

**Authors:** Margarita Pustovalova, Polina Pugacheva, Natalia Vorobyeva, Nelly Babayan, Anna Chigasova, Andrey Osipov, Anzhela Sargsyan, Gohar Tadevosyan, Ruzanna Grigoryan, Natalya Sarkisyan, Yuriy Fedotov, Alisa Manukyan, Andrey Tsishnatti, Denis Guryev, Ashot Vardanyan, Rouben Aroutiounian, Galina Hovhannisyan, Sergey Leonov, Andreyan N. Osipov, Bagrat Grigoryan

**Affiliations:** 1 Moscow Center for Advanced Studies, Kulakova Str. 20, Moscow 123592, Russia; pu.margo@mail.ru (M.P.); polina.pugachewa@gmail.com (P.P.); sergey.leonov@yahoo.com (S.L.); 2 State Research Center-Burnasyan Federal Medical Biophysical Center of Federal Medical Biological Agency (SRC-FMBC), Moscow 123098, Russia; nuv.rad@mail.ru (N.V.); annagrekhova1@gmail.com (A.C.); ufedotov@mail.ru (Y.F.); tsishnatti96@mail.ru (A.T.); denis.guryev@gmail.com (D.G.); 3 N.N. Semenov Research Center of Chemical Physics, Russian Academy of Sciences, Moscow 119991, Russia; a-2-osipov@yandex.ru; 4 Institute of Molecular Biology NAS RA, 7 Hasratyan, Yerevan 0014, Armenia; n_babayan@mb.sci.am (N.B.); g_tadevosyan@mb.sci.am (G.T.); r_grigoryan@mb.sci.am (R.G.); n_sarkisyan@mb.sci.am (N.S.); alisamanukyan77737@gmail.com (A.M.); 5 Research Institute of Biology, Yerevan State University, 1 Manoogian, Yerevan 0025, Armenia; angela.sargsyan@ysu.am (A.S.); rouben_a@hotmail.com (R.A.); galinahovhannisyan@ysu.am (G.H.); 6 CANDLE Synchrotron Research Institute, 31 Acharyan, Yerevan 0040, Armenia; vardanyan@asls.candle.am (A.V.); grigory@asls.candle.am (B.G.); 7 Emanuel Institute for Biochemical Physics, Russian Academy of Sciences, Moscow 119991, Russia

**Keywords:** laser-driven accelerators, ionizing radiation, radiotherapy, ultrashort pulsed electron beam, DNA double-strand breaks, γH2AX, non-small cell lung cancer, transcriptomics

## Abstract

Laser-driven accelerated particle beams have significantly advanced cancer treatment by facilitating the delivery of exceptionally high dose rates of radiation to solid tumors within femto- to picosecond timescales. This investigation compared the radiobiological effectiveness of ultrashort pulsed electron beams, generated by the Advanced Research Electron Accelerator Laboratory (AREAL) accelerator, with conventional X-rays on two non-small cell lung cancer (NSCLC) cell lines: A549 (wild-type p53) and H1299 (p53-deficient). NSCLC cells were irradiated using either the AREAL accelerator (with a peak dose rate of 1.6 × 10^10^ Gy/s, a pulse duration of 4.5 × 10^−13^ s, and a repetition rate of 20 Hz) or an X-ray unit at an absorbed dose rate of 0.85 Gy/min. Clonogenic survival analysis, γH2AX foci enumeration, and genome-wide transcriptome analysis were conducted. Clonogenic survival curves showed increased radiosensitivity of A549 cells following AREAL exposure compared to X-rays (RBE = 1.2), whereas H1299 radiosensitivity remained unchanged. In both cell lines, AREAL exposure resulted in a greater dose-dependent accumulation of residual γH2AX foci 24 h after irradiation than conventional X-rays, suggesting more persistent DNA damage signaling. Transcriptomic analyses revealed broader gene expression changes after AREAL irradiation and suggested distinct p53-related responses. Pathway-level analysis demonstrated that A549 cells exhibited reduced DNA repair activity, accompanied by dysregulation of cell cycle progression and apoptosis, whereas H1299 cells displayed transcriptomic signatures consistent with enhanced homologous recombination activity. Overall, these findings indicate that ultrashort pulsed electron beams induce p53-related responses distinct from those of conventional X-rays and warrant further investigation of this technology as a potential radiotherapy modality.

## 1. Introduction

Lung cancer is the leading cause of cancer-related mortality worldwide, with approximately 85% of all diagnosed cases classified as non-small cell lung cancer (NSCLC) [1]. Radiotherapy serves as a primary treatment modality for locally advanced unresectable tumors, either alone or administered concomitantly with chemotherapy. Despite recent advancements in image-guided technologies, the efficacy of radiotherapy remains constrained by the therapeutic window, the delicate balance between delivering a lethal dose to tumor cells and preserving the tolerance of surrounding normal tissues [2].

High-dose-rate radiotherapy techniques, including laser-driven particle accelerators, have emerged as a promising cancer treatment strategy. Ultrashort-duration (from hundreds of femtoseconds to picoseconds) radiation delivered at ultra-high instantaneous dose rates (up to tens of GGy/s during a pulse) has been shown to reduce normal tissue toxicity commonly associated with conventional radiotherapy while maintaining local tumor control [3,4,5]. Mechanistically, this phenomenon relies on the fact that the duration of a single ultrashort pulse is significantly shorter than the half-life (t_1/2_) of the free radicals (e.g., hydroxyl radicals with t_1/2_ ≈ 1 ns) responsible for inducing DNA damage in normal tissues [6]. Conversely, the ultra-high peak dose rate of laser-driven electron beams invokes a dose-rate effectiveness factor (DREF), where high energy deposition within cancer cells occurs concurrently with primary radical reactions, multiple biomolecular lesions (e.g., membrane and DNA damage), and subsequent repair processes. However, radiobiological research on ultrashort pulsed radiotherapy remains limited and requires thorough validation before clinical implementation.

This study aimed to analyze the radiobiological effects of ultrashort pulsed electron beams generated by the state-of-the-art Advanced Research Electron Accelerator Laboratory (AREAL) accelerator. The primary objective was to conduct a comprehensive comparison of these beams with conventional X-rays, focusing on their effects on two distinct human NSCLC cell lines: A549 (with the wild-type p53) and H1299 (p53-deficient). Specifically, we evaluated clonogenic survival, residual γH2AX foci kinetics, genome-wide gene expression patterns, and the activation levels of crucial cellular pathways, including DNA repair, cell-cycle arrest, and cell death.

## 2. Results

### 2.1. Clonogenic Assay of NSCLC Cell Lines After Treatment with Laser-Driven Ultrashort Electron Beams and X-Rays

**Figure 1:**
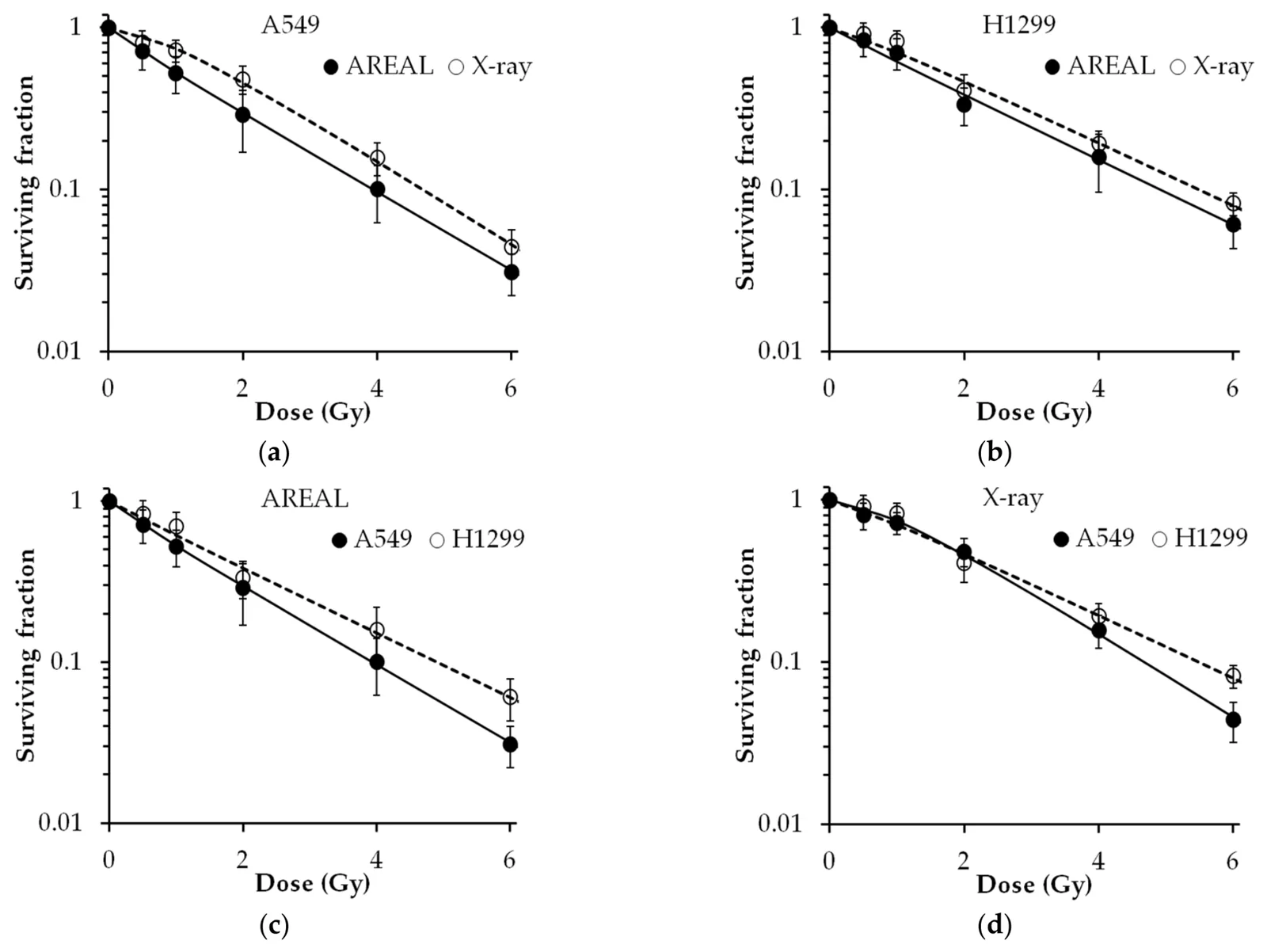
Survival curves for A549 (**a**) and H1299 (**b**) cells exposed to ultrashort electron beams (AREAL) or X-rays. Direct comparison of the survival curves between the two NSCLC cell lines after AREAL (**c**) or X-ray irradiation (**d**). Data represent the mean ± SD from three independent experiments. The surviving fraction is plotted on a logarithmic scale.

Clonogenic survival analysis [7] was performed to compare the radiobiological effectiveness (RBE) of AREAL ultrashort electron beams and conventional X-rays in A549 and H1299 NSCLC cell lines (Figure 1). The RBE of the ultrashort electron beams was calculated at a surviving fraction (SF) of 0.1. At this 10% survival level, AREAL irradiation was significantly more effective in reducing the survival of A549 cells compared to X-rays, yielding an RBE of 1.2. Conversely, no differences were observed between the test and reference radiation modalities for H1299 cells, resulting in an RBE of 1.0. Notably, H1299 cells demonstrated a trend toward overall lower radiosensitivity compared to A549 cells after both AREAL and X-ray exposures (Figure 1c,d), though this difference did not reach statistical significance.

While the X-ray survival curves exhibited the typical shoulder, cell survival following ultrashort electron beam irradiation demonstrated a steeper dose-dependent decline and was best fitted by a linear model. For A549 cells, the average linear (α) and quadratic (β) parameters for X-rays were 0.31 Gy^−1^ and 0.03 Gy^−2^, respectively, whereas the α parameter for the ultrashort electron beams was 0.58 Gy^−1^. For H1299 cells, the α and β parameters for X-rays were estimated at 0.38 Gy^−1^ and 0.01 Gy^−2^, respectively, while the α parameter for the ultrashort electron beams was 0.47 Gy^−1^.

### 2.2. Dose–Responses for Residual γH2AX Foci

**Figure 2:**
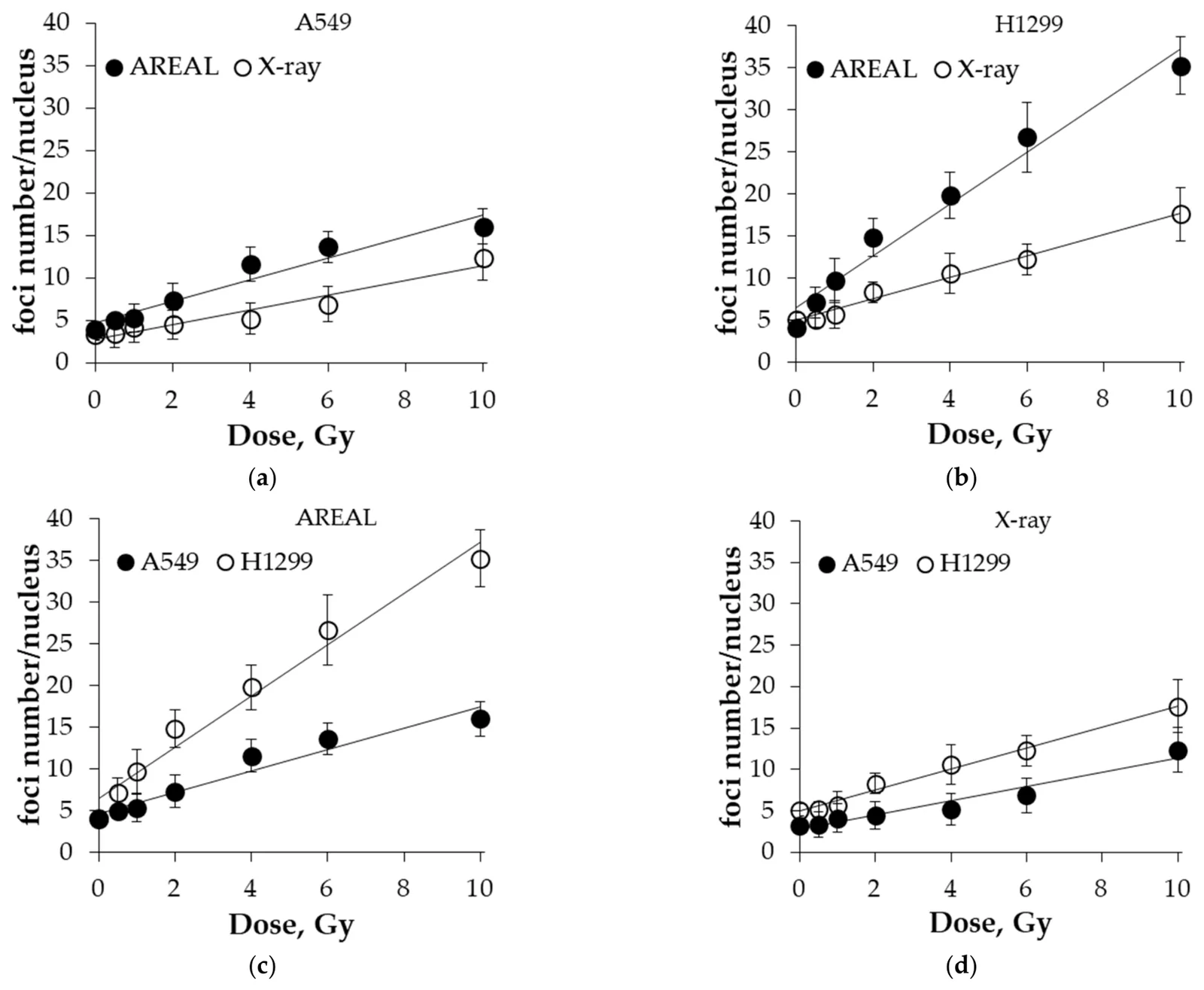

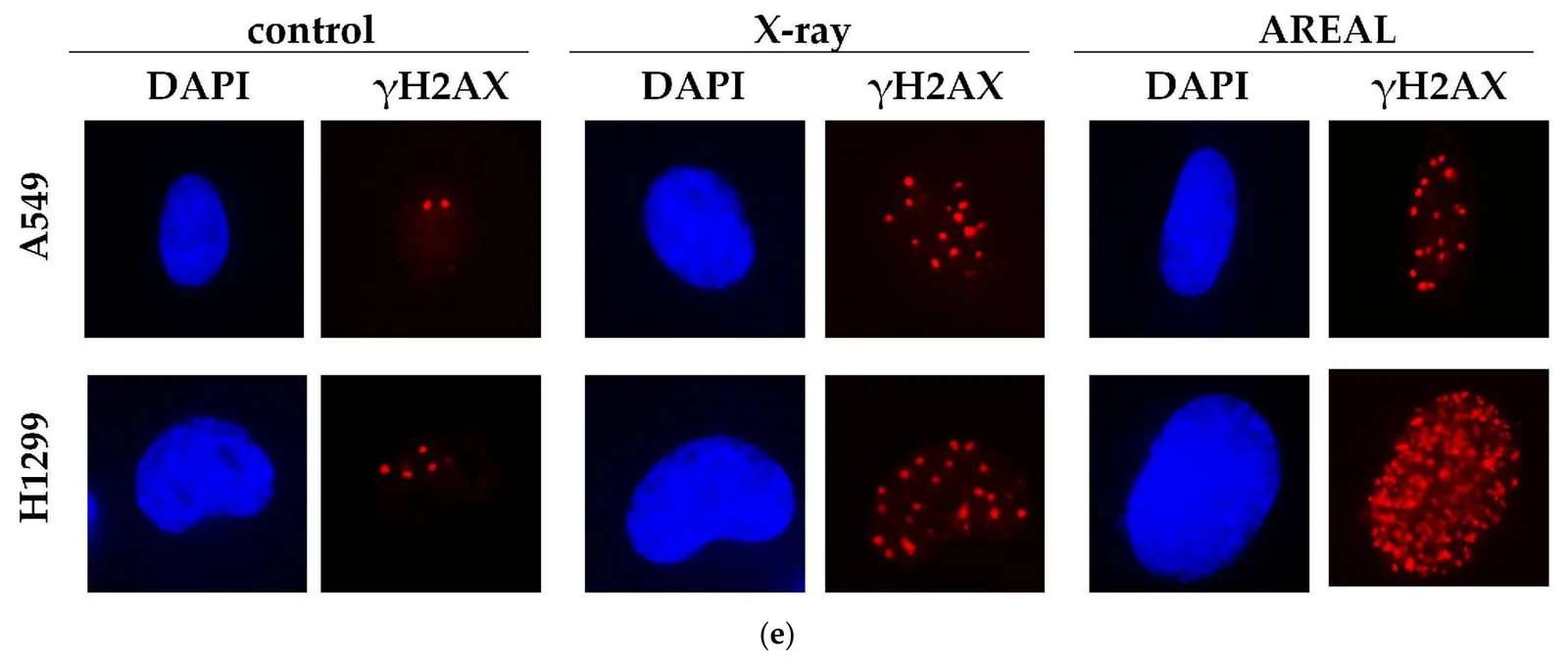
Dose-dependent changes in the number of residual γH2AX foci in A549 (**a**) and H1299 (**b**) NSCLC cells exposed to ultrashort electron beams (AREAL) or X-rays. Direct comparison of the foci kinetics between the two NSCLC cell lines after AREAL (**c**) or X-ray (**d**) irradiation. Representative fluorescence microscopy images of DAPI-stained nuclei and γH2AX foci in control cells and cells exposed to X-rays or AREAL radiation are shown in panel (**e**). Images were acquired 24 h post-irradiation at a dose of 10 Gy (60× objective).

Both cell lines exhibited distinct dose–response profiles for residual γH2AX foci 24 h post-irradiation, which served as the second radiobiological endpoint. Consistent with the clonogenic assay findings, the ultrashort pulsed electron beam (AREAL) irradiation induced a pronounced and statistically significant dose-dependent increase in the number of residual γH2AX foci. This effect was significantly higher than that observed for conventional X-rays in both A549 (Z = 2.4, *p* = 0.016) and H1299 cells (Z = 12.2, *p* < 0.0001) (Figure 2a,b). The dose–response relationships were best described by a linear equation (y = a + bx), where the slope coefficient “*b*” represents the incremental increase in foci per unit dose. A comparative analysis of these slope coefficients across the different exposure conditions enabled the quantification of the fold-difference in DNA damage response per unit dose between the two radiation modalities.

The changes in the number of residual foci were described by the following linear equations: (1) and (2) for A549 cells following AREAL and X-ray exposure, respectively, and (3) and (4) for H1299 cells under the same respective conditions.

(1) y = (1.27 ± 0.14) × x + (4.70 ± 0.68), R^2^ = 0.94

(2) y = (0.86 ± 0.10) × x + (2.80 ± 0.46), R^2^ = 0.94

(3) y = (3.06 ± 0.22) × x + (6.52 ± 1.06), R^2^ = 0.97

(4) y = (1.27 ± 0.07) × x + (4.98 ± 0.31), R^2^ = 0.99,

where “y” is the mean number of foci per cell nucleus and “x” is the irradiation dose (Gy).

Notably, H1299 cells, despite exhibiting lower overall radiosensitivity, displayed approximately twofold higher numbers of residual γH2AX foci compared to A549 cells following both AREAL exposure (Z = 6.7, *p* < 0.0001) and X-ray irradiation (Z = 3.5, *p* = 0.0004) (Figure 2c,d). This apparent discrepancy aligns with previous studies utilizing panels of tumor and normal cell lines, which demonstrated that initial γH2AX levels, foci-resolution kinetics, and residual γH2AX signals do not always reliably predict clonogenic survival [8,9].

These results clearly indicate that NSCLC cells exposed to AREAL ultrashort-pulsed electron beams exhibit a significantly higher number of residual γH2AX foci 24 h post-irradiation than those exposed to X-rays. This finding suggests either greater persistence of DNA damage or a slower resolution of DNA damage signaling following ultrashort-pulsed exposure. This observation further highlights the potential of AREAL as an effective radiotherapy modality for NSCLC treatment, particularly for tumors where conventional X-ray irradiation yields limited efficacy.

### 2.3. Differential Gene Expression in NSCLC Cells Following Ultrashort-Pulsed Beam (AREAL) and X-Ray Irradiation

**Figure 3:**
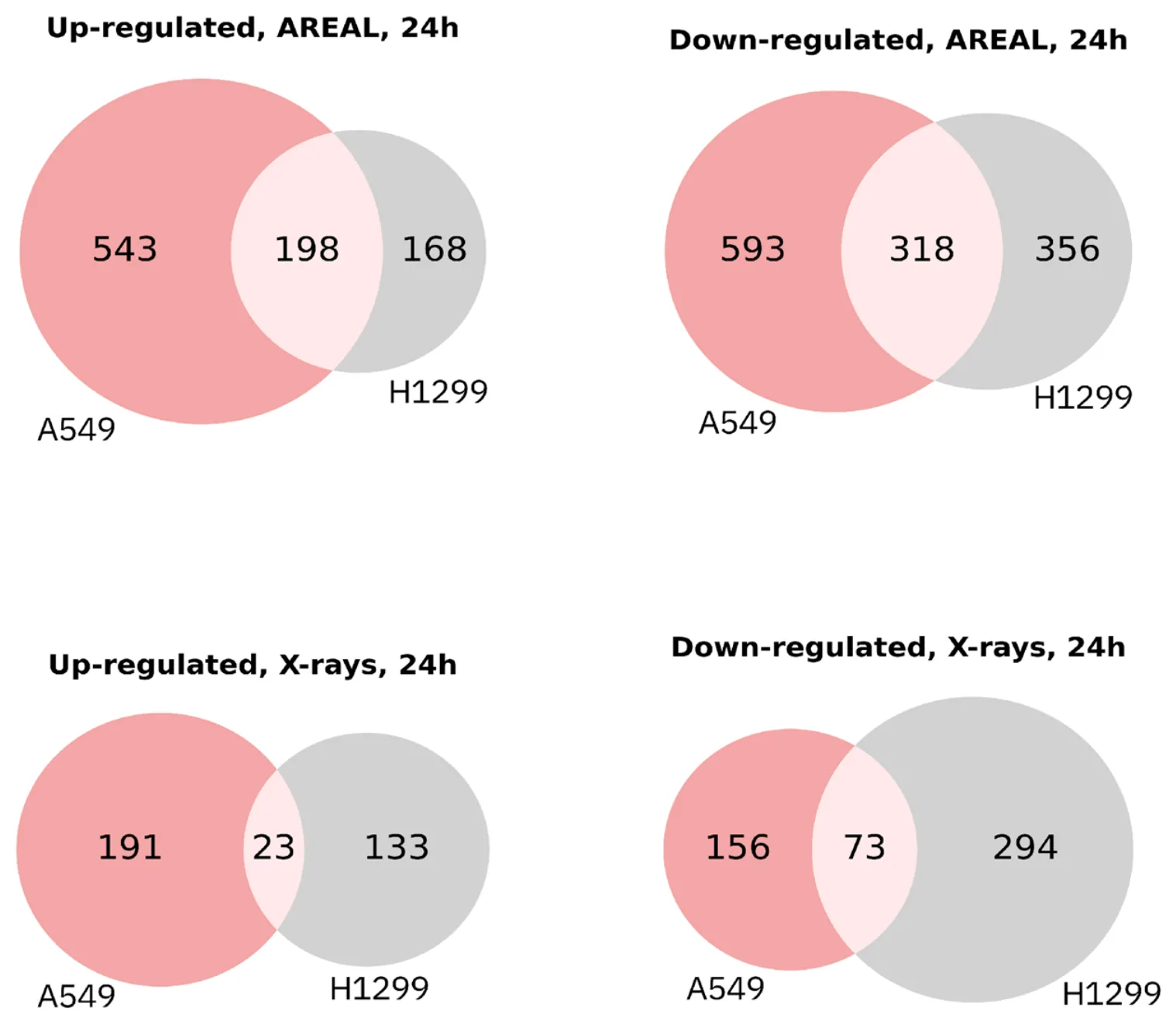
Venn diagrams showing the numbers of unique and shared differentially expressed genes (DEGs) in A549 and H1299 cells 24 h following AREAL or X-ray irradiation.

To evaluate gene expression changes between the two NSCLC cell lines, RNA sequencing (RNA-seq) was performed 24 h post-irradiation. Both AREAL and X-ray exposures significantly increased the number of differentially expressed genes (DEGs; defined as log10(control) > 1 and |log2FC| > 1) in both the A549 and H1299 cell lines (Figure 3). Among these, 198 genes were commonly up-regulated and 318 genes were down-regulated in both A549 and H1299 cells 24 h after AREAL exposure (Figure 3). In contrast, X-ray irradiation up-regulated only 23 and down-regulated 73 common genes across the two cell lines (Figure 3).

**Figure 4:**
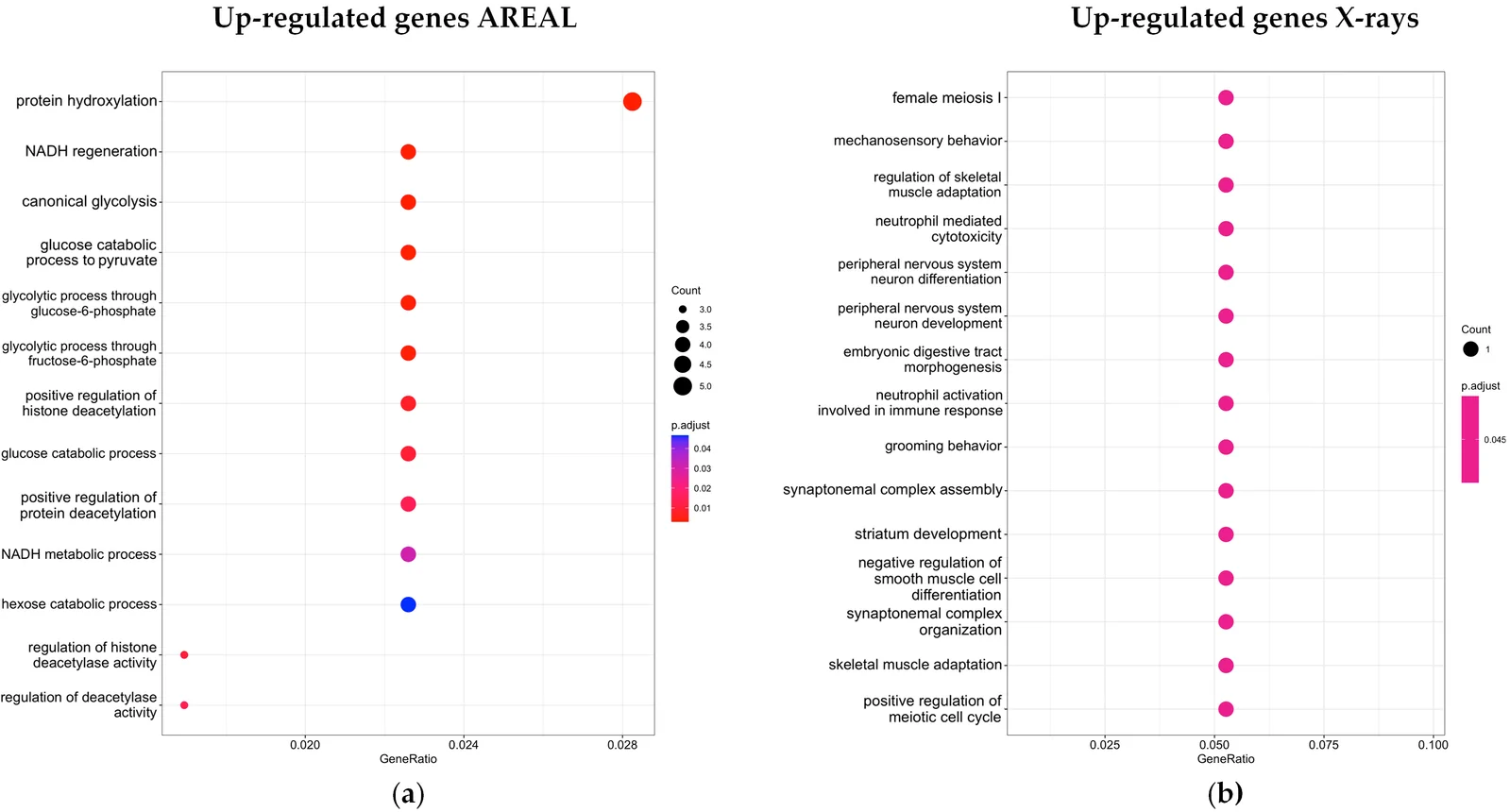

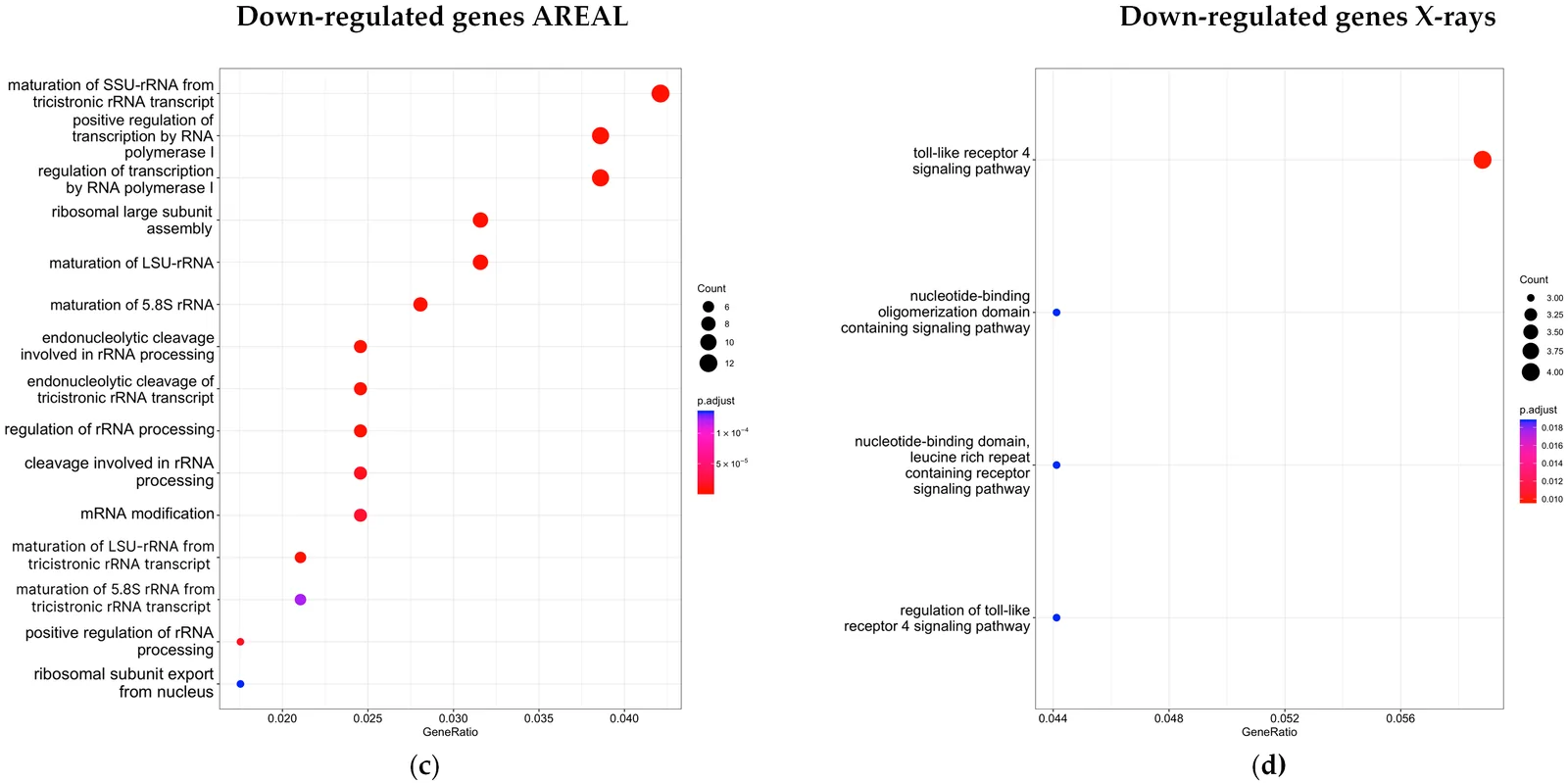
Gene Ontology (GO) biological process enrichment analysis of commonly deregulated genes in A549 and H1299 cells 24 h following AREAL or X-ray irradiation. Panels show commonly up-regulated genes after AREAL (**a**) and X-ray (**b**) exposure, and commonly down-regulated genes after AREAL (**c**) and X-ray (**d**) exposure. Statistical significance was defined at an adjusted *p*-value < 0.05.

Gene Ontology (GO) enrichment analysis of the commonly deregulated genes [10] revealed that terms related to protein hydroxylation, glucose metabolism, and protein/histone modification were overrepresented among the up-regulated genes following AREAL exposure (Figure 4a). In contrast, X-ray induced common changes were enriched for terms associated with muscle development, neurological development, meiosis, and neutrophil activation. These GO categories indicate the potential involvement of cytoskeletal remodeling, mechanotransduction, and DNA recombination pathways, although they should be interpreted as indicative trends rather than direct evidence of pathway regulation (Figure 4b). Ribosomal RNA biosynthesis was predominantly down-regulated in both cell lines after AREAL exposure (Figure 4c), whereas X-rays significantly reduced the activation of the Toll-like receptor 4 (TLR4) pathway (Figure 4d).

### 2.4. Pathway Activation Level (PAL) Analysis of Radiation-Induced Transcriptomic Changes in NSCLC Cells

**Figure 5:**
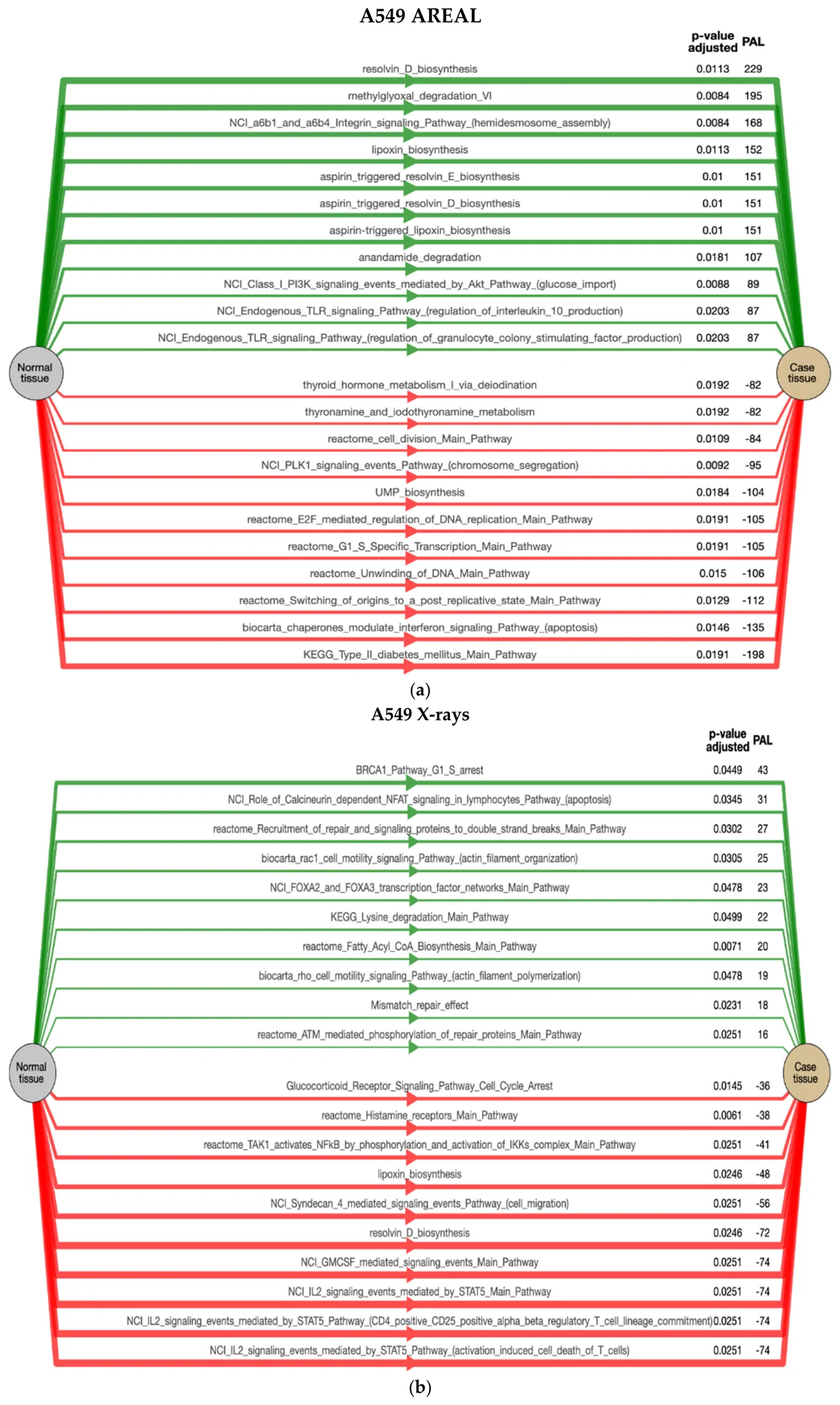

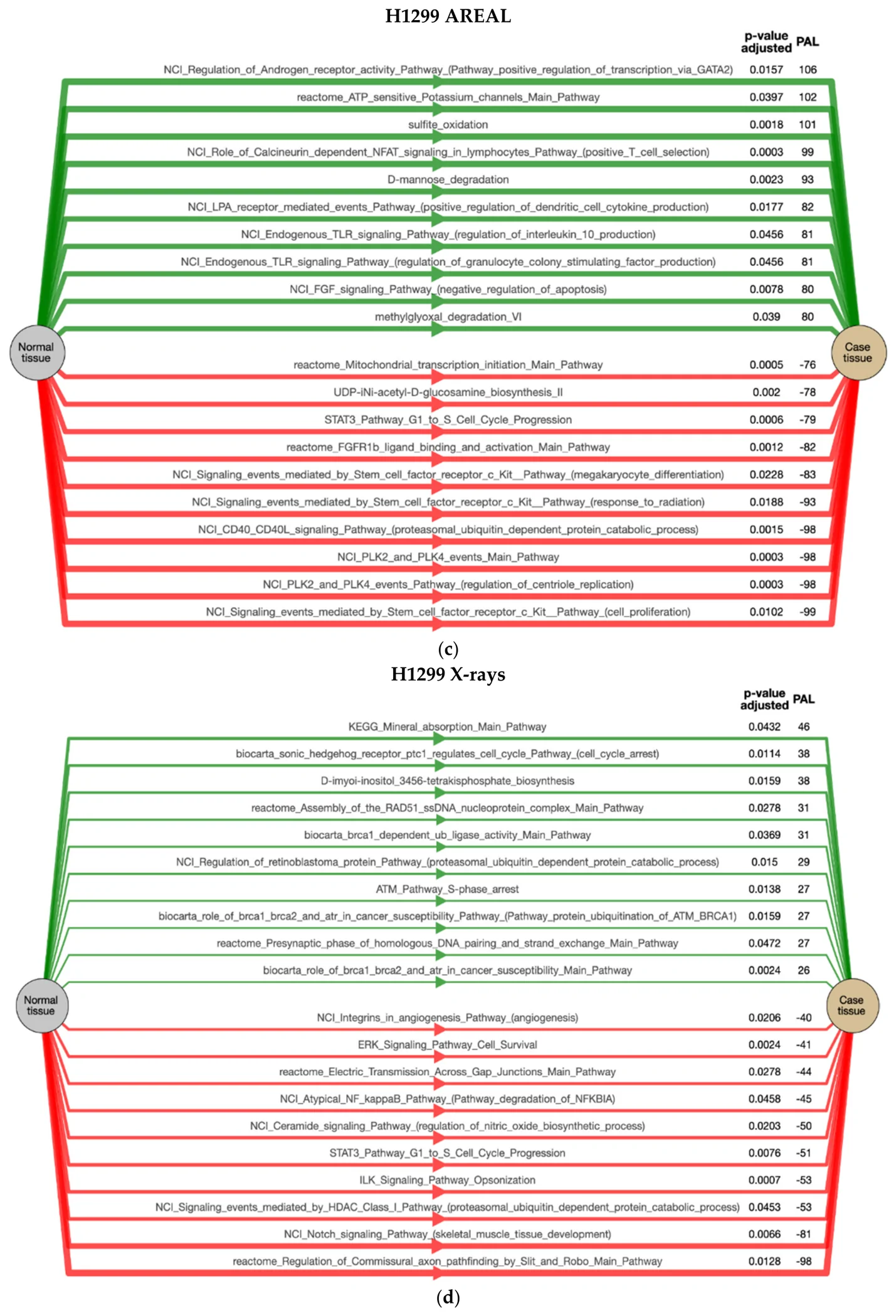
Pathway Activation Level (PAL) analysis of transcriptomic pathway profiles in A549 and H1299 cells. The top 10 up- (green lines) and down-regulated (red lines) pathways are shown 24 h following exposure to ultrashort pulsed electron beams (AREAL) or X-rays in A549 (**a**,**b**) and H1299 (**c**,**d**) cells. Statistical significance was determined using the Benjamini–Hochberg adjusted *p*-value < 0.05 (only pathways containing 10 or more genes are shown).

To evaluate functional transcriptomic profiles, Pathway Activation Level (PAL) analysis was performed to compare pathway activation states between irradiated and unirradiated control A549 and H1299 cells. PALs represent logarithmic fold-change expression levels (case compared to control samples) for gene products involved in a specific molecular pathway [11]. The ten most up-regulated (PAL > 0) and down-regulated (PAL < 0) pathways identified following AREAL and X-ray irradiation are shown in Figure 5a–d. Comprehensive PAL values along with their corresponding adjusted *p*-values for all significantly modulated pathways across both cell lines and irradiation modalities are provided in Supplementary Table S1.

In A549 cells, PAL analysis revealed the modulation of several pathways related to the generation of anti-inflammatory mediators, including anandamide degradation, AKT-mediated glucose import, and the TLR pathway involved in regulating interleukin-10 (IL-10) and granulocyte colony-stimulating factor (G-CSF) production. These findings suggest a complex interplay between the metabolic, immunological, and inflammatory responses during the radiation reaction of A549 cells exposed to AREAL. Furthermore, the A549 response to AREAL exposure involved several other crucial pathways associated with thyroid hormone metabolism, cell division, and chromosome segregation, as well as DNA transcription, replication, and unwinding (Figure 5a).

Additionally, PAL analysis highlighted a significant down-regulation of pathways related to apoptosis and type 2 diabetes mellitus; in lung cancer cell lines, the latter reflects shifts in insulin and energy-metabolism signaling. These processes ranked among the top 10 pathways with the lowest PAL values. In contrast, X-ray irradiation induced significant changes in DNA repair, cell cycle arrest, cytoskeletal reorganization, cell motility, FOXA2/FOXA3 activation, and apoptotic pathways in A549 cells (Figure 5b). Concurrently, the biosynthesis of resolvins and lipoxins, as well as the production of granulocyte colony-stimulating factor (G-CSF), were significantly down-regulated.

These findings reveal that the transcriptional responses of A549 cells to AREAL and X-ray irradiation differ markedly regarding the direction of activation across several pathway-related gene sets. However, these observed trends should be interpreted as qualitative indications of divergence rather than statistically proven opposite effects.

In H1299 cells, exposure to AREAL led to a pronounced increase in the activity of the androgen receptor pathway, which is mediated by the transcription factor GATA-binding protein 2 (GATA2). This activation correlated with several key biological processes, such as ATP-sensitive K^+^ channel regulation, sulfite oxidation, D-mannose degradation, and the negative regulation of apoptosis. Furthermore, this axis played a significant role in regulating cellular defense systems in this p53-deficient cell line. Exposure to AREAL also resulted in a substantial down-regulation of various pathways, including mitochondrial transcription, cell cycle progression (specifically the STAT3-mediated G_1_/S progression, as well as PLK2 and PLK4 signaling events), the c-Kit pathways, and the FGFR1b pathway (Figure 5c). These findings indicate that AREAL exposure exerts complex, widespread effects on cellular processes. Simultaneously, pathway up-regulation in X-ray-irradiated H1299 cells was predominantly associated with mineral absorption (such as inositol polyphosphate) and DNA repair. Notably, homologous recombination (HR) pathways, including RAD51 assembly and BRCA1/BRCA2 signaling, were up-regulated, which aligns with the activation of S and G_2_/M cell cycle arrest driven by patched 1 (PTC1) receptor and ATM kinase activities (Figure 5d). Conversely, the SLIT/ROBO pathway and Notch signaling pathways were among the most strongly down-regulated following X-ray exposure in H1299 cells. In these cells, crucial intercellular interactions (including integrin-mediated angiogenesis and electrical transmission across gap junctions), as well as pivotal signaling cascades (such as ILK, ERK, and STAT3 transduction pathways) were predominantly suppressed. Additionally, the regulation of NO biosynthesis by ceramides and the degradation of NFKBIA also exhibited down-regulation in these cells (Figure 5d).

### 2.5. DNA Repair Pathway Activation Levels in NSCLC Cells Following Ultrashort-Pulsed Beam (AREAL) and X-Ray Irradiation

**Figure 6:**
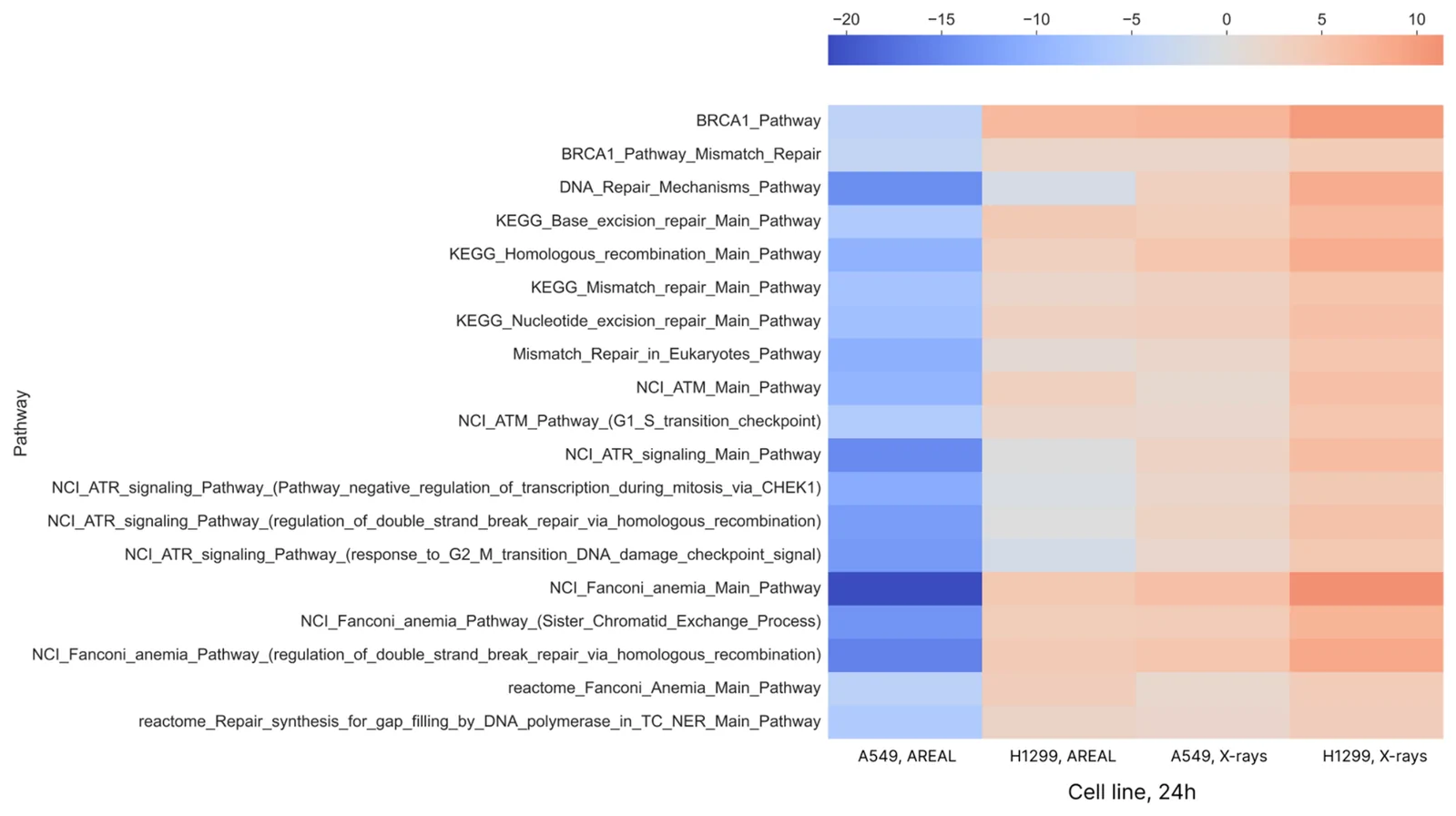
Heatmap of selected DNA repair pathways in A549 and H1299 cells 24 h following AREAL or X-ray irradiation. PAL values for both irradiation types are adjusted to the unirradiated control, and AREAL PAL values are normalized to X-rays (with X-ray irradiated cells used as a reference).

To evaluate variations in DNA repair pathway activity between the A549 and H1299 cell lines following AREAL or X-ray exposure, PAL values were analyzed for a subset of 19 filtration-validated repair pathways. These pathways were predominantly associated with single-strand break repair (such as base excision repair and nucleotide excision repair) and homologous recombination (HR) mechanisms (Figure 6).

Both A549 and H1299 cells exhibited a modest increase in DNA repair activity 24 h post-irradiation with X-rays (PAL values ≤ 10). Notably, the activation of the BRCA1 and Fanconi anemia pathways in H1299 cells following X-ray exposure demonstrated a robust profile with PAL values approaching 10, thereby indicating enhanced DNA double-strand break repair activity via HR. Compared with the X-ray reference control, AREAL-exposed H1299 cells exhibited a slight decrease in DNA repair activity across the evaluated pathways (Figure 6). This attenuation of DNA repair PAL scores was even more pronounced in A549 cells than in H1299 cells. The pathways exhibiting the most substantial down-regulation included HR-mediated by the Fanconi anemia pathway, alongside the ATR, ATM, and mismatch repair cascades (Figure 6).

### 2.6. Activation Profiles of Cell-Cycle and Cell-Death Pathways in NSCLC Cells Following Ultrashort-Pulsed Beam (AREAL) and X-Ray Irradiation

To further elucidate the transcriptomic alterations between AREAL and X-ray exposed NSCLC cells, functional signaling pathways associated with cell-cycle regulation and cell-death modalities were evaluated.

**Figure 7:**
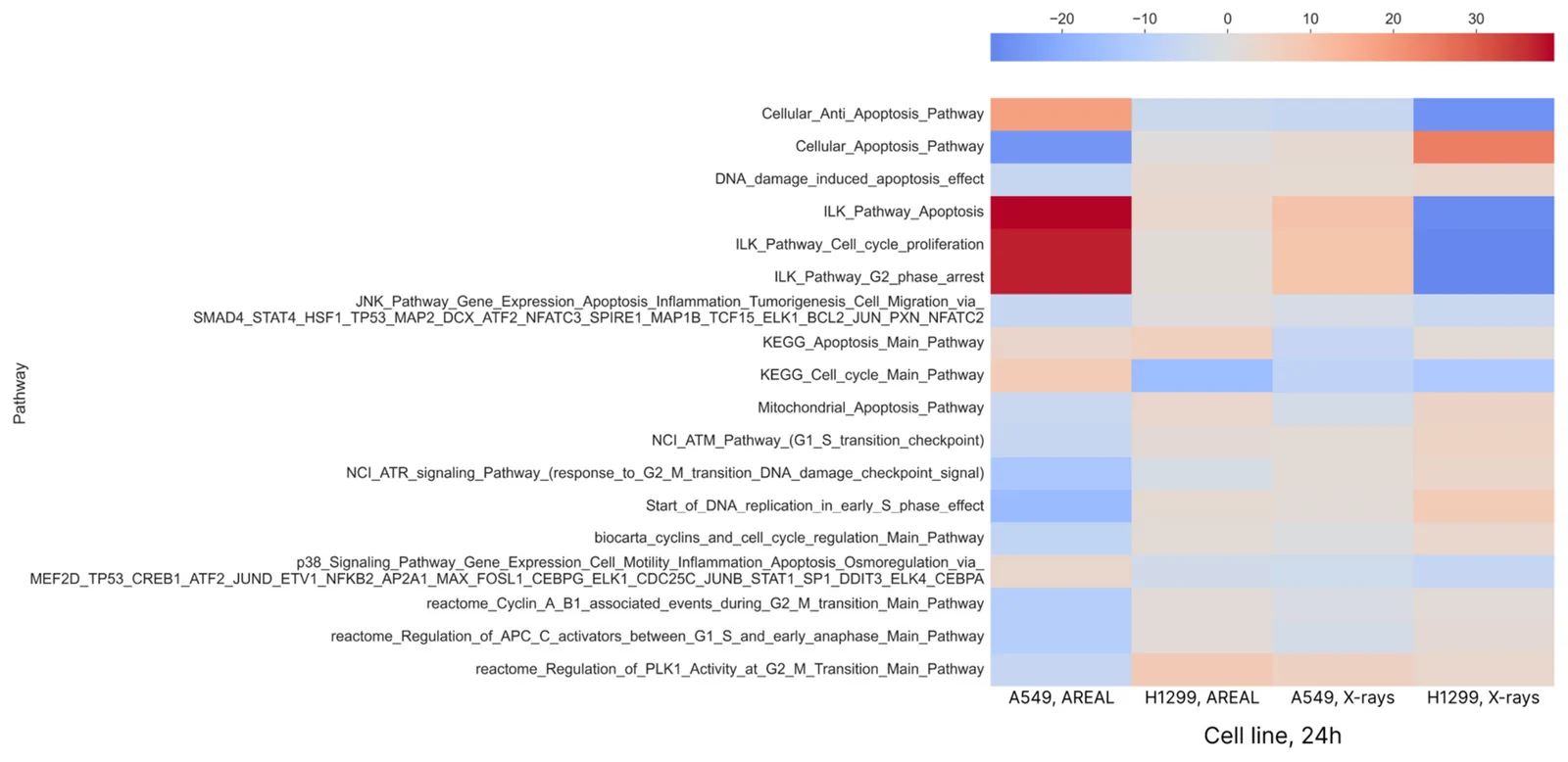
Heatmap of selected cell cycle and cell death pathways in A549 and H1299 cells 24 h following AREAL or X-ray irradiation. PAL values for both irradiation types are adjusted to the unirradiated control, and AREAL PAL values are normalized to X-rays (with X-ray-irradiated cells used as a reference).

**Figure 8:**
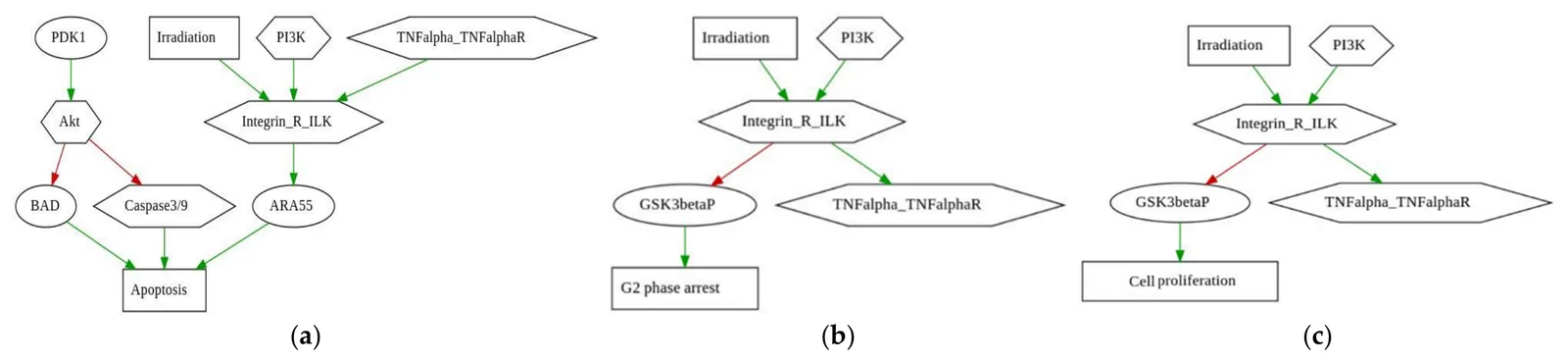
Schematic representation of the integrin-linked kinase (ILK) signaling pathway involved in (**a**) apoptosis, (**b**) G_2_-phase arrest, and (**c**) cell proliferation in A549 cells following AREAL irradiation. Arrows show molecular interactions within a pathway: green stands for activation, red for inhibition.

Compared to X-ray-irradiated cells, AREAL-exposed A549 cells demonstrated a robust activation profile (with PAL values exceeding 30) for pathways governing integrin-linked kinase (ILK) signaling, G_2_-phase arrest, and cell proliferation (Figure 7). Notably, anti-apoptotic regulation in these cells was achieved through 3-phosphoinositide-dependent protein kinase 1 (PDK1)/Akt-mediated inactivation of BAD and caspases 3 and 9, whereas apoptosis was concomitantly induced via the ILK/ARA55 pathway (Figure 8a). Furthermore, the ILK/GSK-3β interaction was involved in modulating cell-cycle progression and proliferation following AREAL irradiation of A549 cells (Figure 8b,c). The same pathways, though to a lesser extent, were found to be activated following X-ray exposure compared to the unirradiated control (Figure 8a).

Figure 7 demonstrates a marked contrast between the impact of X-ray irradiation on apoptotic and cell-cycle PAL values in H1299 cells and the effects observed in A549 cells following AREAL exposure. Specifically, G_2_/M transition pathways in H1299 cells were significantly up-regulated 24 h post-irradiation with AREAL, particularly via the “Reactome Regulation of PLK1 activity at G_2_/M transition” pathway. Conversely, the “KEGG Cell cycle” pathway exhibited a pronounced down-regulation, with PAL values approaching −10 compared with the unirradiated control.

**Figure 9:**
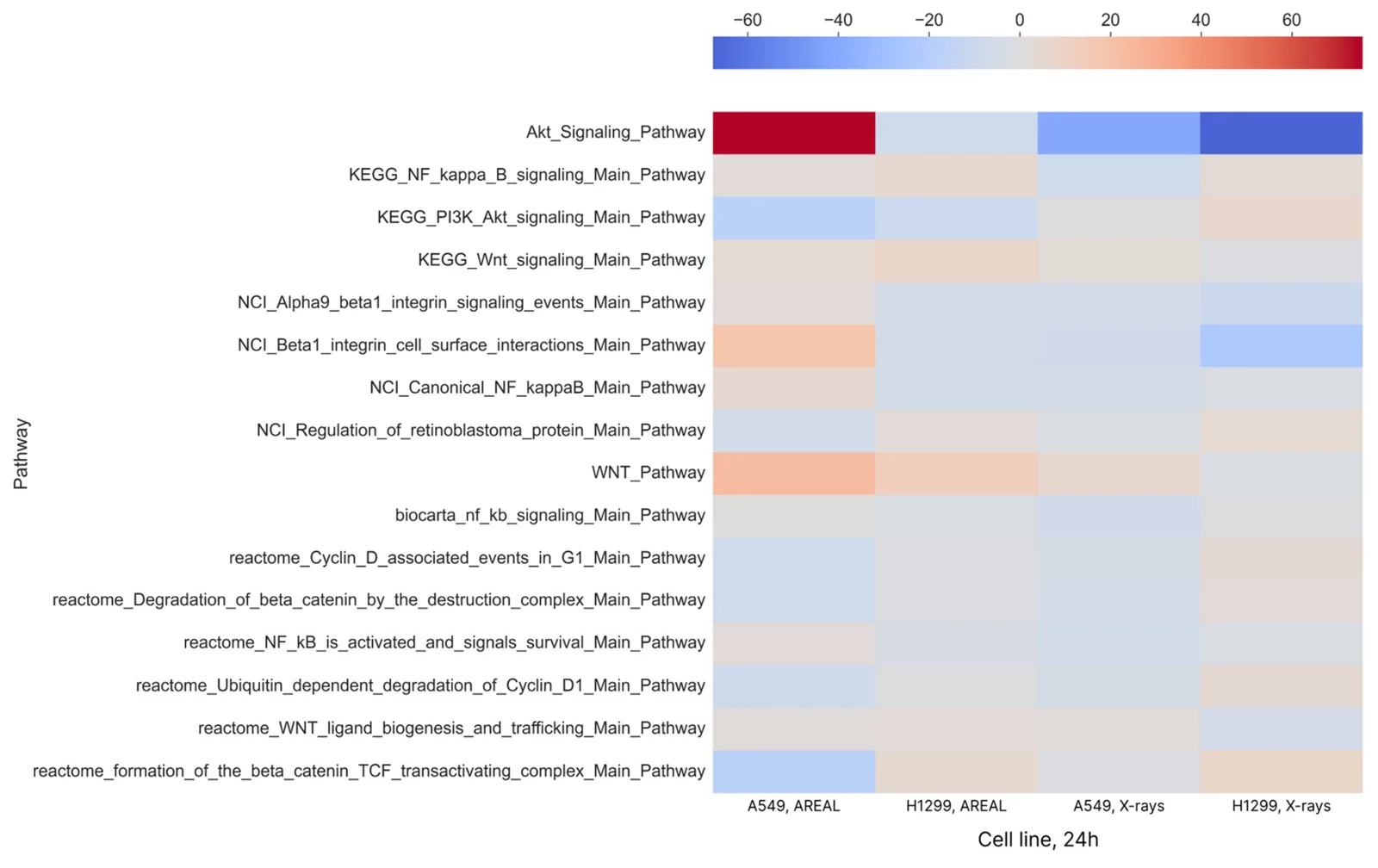
Heatmap of RNA-seq-based transcriptomic analysis for 16 selected pathways in A549 and H1299 cells. PAL values for both irradiation types are adjusted to the unirradiated control, and AREAL PAL values are normalized to X-rays (with X-ray-irradiated cells used as a reference).

The involvement of key signaling pathways associated with cancer radioresistance, such as AKT, NF-kB, WNT, p53 and β-catenin cascades (16 pathways in total), was further investigated (Figure 9). X-ray exposure resulted in a decrease in AKT and β-integrin signaling in both NSCLC cell lines. Notably, the AREAL-exposed A549 cells showed elevated activation levels of the AKT, β-integrin, and WNT pathways, which correlates with ILK signaling. In contrast, the WNT pathway was only minimally activated in H1299 cells. NF-kB, β-catenin, and cyclin D1 signaling activities exhibited minimal to no alteration when comparing AREAL-exposed cells to their X-ray-irradiated counterparts.

## 3. Discussion

Laser-driven particle accelerators have emerged within the context of the FLASH radiotherapy approach as a promising strategy for cancer treatment following the landmark publication by Favaudon et al., which demonstrated that ultra-high-dose-rate (≥40 Gy s^−1^) electron beams produced fewer pulmonary lesions in C57BL/6 mice than conventional dose-rate exposure [12]. Although the use of ultra-high instantaneous pulse dose rates (up to tens of GGy/s) and ultrashort (femtosecond- to picosecond-range) delivery represents a promising modality, conflicting evidence remains regarding whether laser-generated electron beams exert a differentially significant impact on tumor cells [13].

In this study, we assessed the relative biological effectiveness (RBE) of laser-generated ultrashort pulsed electron beams by focusing on the clonogenic survival of two NSCLC cell lines, thereby examining the impact of ultra-high peak dose rates resulting from ultrashort pulse duration. An enhanced RBE with respect to cell survival for pulsed laser-generated electrons compared with X-rays was observed exclusively in A549 cells (RBE = 1.2). In H1299 cells, no significant difference in surviving fractions was detected between the laser-generated pulsed electron beam and X-ray exposures. Across both NSCLC cell lines, H1299 (p53-deficient) consistently demonstrated a trend toward lower overall radiosensitivity compared to the A549 (wild-type p53) cell line, regardless of the irradiation modality.

Although A549 cells, in general, exhibited higher radiosensitivity, they demonstrated lower levels of residual γH2AX foci following both AREAL and X-ray exposure when compared to H1299 cells. A possible explanation for this discrepancy between foci numbers and clonogenic survival is that the remaining foci might not always signify unrepaired double-strand breaks (DSBs); instead, they may reflect delayed dephosphorylation of γH2AX. Moreover, this disparity might also reflect areas of chromatin decondensation, which can trigger p53-mediated, senescence-like growth arrest [14,15]. These markers may also be generated as secondary foci post-irradiation (post-IR) by the attempted repair of clustered damage sites by DNA glycosylases [16,17]. Furthermore, active HR pathways can lead to the formation of additional γH2AX foci in H1299 cells.

Considering the differences in radiosensitivity and residual foci numbers between the wild-type p53 (A549) and p53-deficient (H1299) NSCLC cell lines, it can be hypothesized that the response of cancer cells to ultrashort pulsed electron beams is specific to their genotype. To explore this hypothesis, a genome-wide transcriptomic analysis was conducted on both cell lines 24 h post-exposure to a 2 Gy dose of either AREAL or X-rays.

The up-regulation of HR-associated pathways (such as the BRCA1 and Fanconi anemia cascades) based on PAL scores in H1299 cells 24 h post-exposure to AREAL aligns with a potential delay in dephosphorylation and the consequent formation of additional γH2AX foci. Conversely, these pathways were down-regulated in A549 cells, particularly when compared to the X-ray reference. Furthermore, PAL analysis indicated that 24 h post-irradiation with AREAL, A549 cells exhibited increased activity in pathways associated with cell-cycle regulation, G_2_/M checkpoint control, and apoptosis—most notably the integrin-linked kinase (ILK) pathway. This up-regulation coincided with the suppression of DNA repair processes. In stark contrast, H1299 cells exposed to X-rays demonstrated an opposing trend regarding activation of these pathways. ILK is a major serine-threonine kinase associated with integrins that is widely expressed in a broad range of human tissues [18]. Accumulating evidence indicates that targeting of ILK modulates radioresistance in head and neck and lung cancer cells, while promoting radiosensitization in glioblastoma cells [19,20,21]. ILK interacts with the cytoplasmic tails of β1-, β2-, and β3-integrins, thereby regulating focal adhesion dynamics, cell spreading, and the subcellular architecture of actin stress fibers [22]. Previous research has demonstrated a dual role for ILK in governing cancer cell biology, where it can function as both a proto-oncogene driving proliferation and a contextual tumor suppressor [23]. Consequently, its functional impact spans a wide array of vital biological processes, including cell growth, division, invasion, migration, angiogenesis, and programmed cell death. Mechanistically, ILK overexpression has been reported to inhibit GSK-3β activity and modulate AKT signaling [24]. Because GSK-3β negatively regulates the WNT cascade and growth factor receptor signaling, its inhibition enhances WNT-related transcription, whereas AKT activation promotes cell survival through the inhibition of pro-apoptotic proteins. In the present study, PAL analysis indicated transcriptomic changes in pathways associated with both ILK/ARA55-mediated apoptosis and AKT-mediated anti-apoptotic signaling in AREAL-exposed A549 cells. These findings suggest the potential concurrent involvement of pro- and anti-apoptotic signaling processes, although functional activation of these pathways warrants further experimental validation [25].

In addition to canonical DNA damage response processes, including DNA repair, cell-cycle regulation, and cell death, alternative metabolic and signaling pathways substantially contribute to the cellular radiation response and the development of radioresistance. In the present study, PAL analysis indicated that the androgen receptor (AR) signaling pathway exhibited one of the highest positive PAL values in H1299 cells following AREAL exposure. The involvement of AR signaling in conferring resistance to radiotherapy is well-documented, and AR expression can be directly regulated by GATA2 [26,27,28]. These findings further suggest that AR signaling poses a significant barrier to overcoming radioresistance, highlighting the potential therapeutic value of developing strategies to suppress this axis.

Although laser-generated pulsed electron beams can deliver doses within femtosecond- to picosecond-range intervals, thereby potentially modifying reactive oxygen species (ROS) kinetics, PAL analysis of AREAL-exposed A549 cells indicated increased transcriptomic activity of pathways associated with inflammation resolution. Chronic inflammation and oxidative damage are closely linked components of the cellular response to ionizing radiation [29]. In A549 cells, the increased PAL values of resolvin- and lipoxin-biosynthesis pathways may therefore represent a potential adaptive or radioprotective response to AREAL irradiation. The resolution of inflammation is an active process regulated by specialized pro-resolving mediators, including lipoxins, E- and D-series resolvins, protectins, neuroprotectins, and maresins [30]. Lipoxins are endogenous arachidonic acid-derived lipid mediators with potent anti-inflammatory properties [31,32]. Previous studies have shown that lipoxins and resolvins can reduce UV-induced inflammation and oxidative stress, as well as irradiation-associated inner-ear damage [33,34,35]. However, their role in mediating responses to laser-generated ultrashort electron beams remains poorly understood and requires further experimental investigation.

Laser-generated ultrashort pulsed electron beam radiotherapy may also play a major role in activating pathways involved in the immune response [36]. Both A549 and H1299 cell lines significantly activated the “Endogenous TLR signaling pathway” involved in regulating interleukin-10 (IL-10) and granulocyte colony-stimulating factor (G-CSF) production following AREAL exposure. Toll-like receptors (TLRs) initiate intracellular signaling pathways leading to the synthesis and secretion of various cytokines and chemokines by cells of the innate immune system, including IL-10 [37]. IL-10 subsequently promotes the activation of tumor-resident CD8^+^ T cells, which can facilitate tumor rejection and the suppression of cancer-associated inflammation [38].

In H1299 cells, the activation of immune-associated pathways induced by AREAL was markedly pronounced, encompassing the enhancement of positive T-cell selection, up-regulation of dendritic cell cytokine production, and accelerated D-mannose degradation. Previously, the role of D-mannose in facilitating the immunotherapy and radiotherapy of triple-negative breast cancer (TNBC) was elucidated [39]. D-mannose can induce abnormal glycosylation and proteasomal degradation of PD-L1, which in turn promotes T-cell activation and subsequent T-cell-mediated cytotoxicity. Moreover, D-mannose-mediated PD-L1 degradation leads to the destabilization of messenger RNA (mRNA) encoding DNA damage repair factors, thereby sensitizing breast cancer cells to ionizing radiation and enhancing the therapeutic efficacy of radiotherapy in murine TNBC models [39]. Remarkably, metformin, a drug widely utilized for the treatment of type 2 diabetes, has also been shown to induce abnormal glycosylation and degradation of PD-L1 [40]. Further investigation is warranted to fully understand the impact of AREAL exposure on tumor-directed immune responses, particularly through the exploration of these dynamics within the complex tumor microenvironment.

This study has several limitations that should be considered when interpreting the results. First, the experiments were performed on only two NSCLC cell lines, which differ not only in p53 status but also in numerous other genetic and epigenetic backgrounds; thus, the observed p53-related effects require validation using isogenic cell lines or targeted p53 knockdown/rescue models. Second, all transcriptomic analyses, including PAL and GO enrichment, are in silico predictions and have not been corroborated by protein-level measurements (e.g., Western blotting), functional DNA repair assays (e.g., HR or NHEJ reporter systems), or direct cell-cycle and apoptosis assays. Third, the dosimetry of the AREAL beam, while carefully performed, involves ultra-high peak dose rates and inherent non-uniform dose distribution, which may affect the reproducibility of cellular responses. Fourth, the clinical relevance of these in vitro findings remains uncertain, as the tumor microenvironment and immune components are absent. Therefore, the proposed mechanisms, such as ILK-mediated apoptosis or AR-driven radioresistance, should be considered hypotheses that warrant further experimental validation before any translational conclusions can be drawn.

## 4. Materials and Methods

### 4.1. Cell Lines

Human NSCLC cell lines H1299 (p53-deficient, ATCC # CRL-5803) and A549 (p53-wild-type, ATCC # CCL-185) were used in this experiment. A549 cells were maintained in DMEM (Gibco, Thermo Fisher Scientific, Waltham, MA, USA, Cat. No. 21331020) whereas H1299 cells were cultured in RPMI 1640 medium (Gibco, Thermo Fisher Scientific, Cat. No. 31870074). Both media were supplemented with 10% fetal bovine serum (FBS, Thermo Fisher Scientific, Cat. No. A5256801), 2.5 mM L-glutamine (Thermo Fisher Scientific, Cat. No. 25030081), 100 IU/mL penicillin (Sigma-Aldrich, Darmstadt, Germany, Cat. No. P3032), and 100 μg/mL streptomycin (Sigma-Aldrich, Cat. No. S9137). The cultures were maintained at 37 °C in a humidified atmosphere containing 5% CO_2_. All experiments were performed using cells in their logarithmic growth phase.

### 4.2. X-Ray and Ultrashort Pulsed Electron Beam Irradiation

Conventional X-ray irradiation was performed at room temperature using a 200 kV RUB RUST-M1 X-ray irradiator facility (Diagnostika-M LLC, Moscow, Russia) at a dose rate of 0.85 Gy/min (operated at 2 × 5 mA with a 1.5 mm Al filter).

Ultrashort pulsed electron beam irradiation was carried out using the laser-driven radiofrequency gun-based linear accelerator at the Advanced Research Electron Accelerator Laboratory (AREAL) [41]. The specific electron beam parameters are presented in Table 1.

Dosimetric measurements to estimate the integral dose over each pulse were performed using a calibrated Faraday cup. Cells were irradiated across a dose range of 0.25–10 Gy (at approximately 140 electron pulses per 1 Gy and a repetition rate of 20 Hz). A peak dose rate of 1.6 × 10^10^ Gy/s was estimated based on an electron pulse duration of 4.5 × 10^–13^ s. The mean absorbed dose rate of 11.70 ± 0.98 Gy/min was calculated over the irradiation period, accounting for a 20 Hz repetition rate, a sample mass of 4.2 g, 1% charge fluctuation and a 1% beam energy fluctuation.

### 4.3. Clonogenic Assay

Cells were seeded into 25 cm^2^ culture flasks and grown to 70–80% confluence before being exposed to a single dose (0, 2, 4, or 6 Gy) of either X-rays or AREAL ultrashort pulsed electron beam irradiation. Immediately following irradiation, cells were harvested by trypsinization and seeded onto 60 mm Petri dishes at a density of 500 (for 0 and 2 Gy) or 2000 (for 4 and 6 Gy) cells per dish. After a two-week incubation period at 37 °C in a humidified atmosphere containing 5% CO_2_, the resulting colonies were fixed with 100% methanol for 15 min at room temperature and subsequently stained with a 0.5% methylene blue solution for 25 min. Colonies containing at least 50 individual cells were counted. The plating efficiency (PE) and surviving fraction (SF) were calculated using the following equations:

(5) $$PE\;=\;\frac{number\;of\;colonies\;formed}{number\;of\;cells\;seeded}\times 100\%$$

(6) $$SF=\;\frac{number\;of\;colonies\;formed\;after\;irradiation}{\left(number\;of\;cells\;seeded\times PE\right)}$$

### 4.4. Calculation of Relative Biological Effectiveness (RBE)

The relative biological effectiveness (RBE) is defined as the ratio of the absorbed dose of the reference radiation (D_ref_, X-rays) to the absorbed dose of the radiation under investigation (D, AREAL electron beams) required to produce the same biological effect (Equation (7)).

(7) $$RBE=\frac{D_{ref}}{D}$$

The dose–response relationships derived from the clonogenic survival dataset were fitted using the linear-quadratic model (LQM) according to Equation (8):

(8) S = exp (−αD − βD^2^),

where the surviving fraction S is a function of the absorbed dose D, while α and β are LQM fitting parameters representing the linear and quadratic components, respectively.

Consequently, by rearranging the LQM to solve for dose, the RBE was evaluated as a function of the surviving fraction S using Equation (9):

(9) $$RBE\;=\;\frac{\alpha \;+\sqrt{\alpha^{2}\;\;-\;\;4\beta lnln\;\left(S\right)\;}\;}{\alpha_{ref}\;+\;\sqrt{{\alpha^{2}}_{ref}\;-\;4\beta_{ref}lnln\;\left(S\right)}}$$

where α, β, α_ref_, and β_ref_ are the linear and quadratic terms of the LQM for the test radiation and the reference photon exposure, respectively.

### 4.5. Immunofluorescence Analysis of γH2AX Foci

Immunofluorescence staining of γH2AX was performed according to a previously described protocol [42]. A primary antibody against γH2AX (dilution 1:200, clone EP854(2)Y, Merck-Millipore, Burlington, VT, USA, Cat. No. MABE205) and secondary goat anti-rabbit IgG (H + L) antibody (Alexa Fluor 488-conjugated, dilution 1:600; Merck-Millipore, Burlington, VT, USA, Cat. No. 16-237) were utilized. Cells were imaged using a Nikon Eclipse Ni-U microscope (Nikon, Tokyo, Japan) equipped with ProgRes MFcool high-resolution camera (Jenoptik AG, Jena, Germany) using filter sets UV-2E/C and B-2E/C. For each data point, a total of 300–400 nuclei were analyzed. Foci quantification was performed using DARFI software (version 2.0, http://github.com/varnivey/darfi; accessed on 19 September 2016).

### 4.6. Transcriptomic Analysis

Exponentially growing A549 and H1299 cells were exposed to a 2 Gy dose either of AREAL or X-ray radiation. Following irradiation, cells were seeded into 6-well plates and incubated in DMEM supplemented with 10% FBS for 24 h. Thereafter, cells were harvested, washed with PBS, and pelleted by centrifugation at 1500 rpm for 10 min at 4 °C. Cells counts were determined, and the cells were collected into PCR tubes via a subsequent centrifugation step under the same conditions, after which the supernatant was discarded completely. The resulting cell pellets were snap-frozen and stored at −80 °C prior to downstream processing. RNA libraries were constructed, sequenced, and initially analyzed following protocols established for the Atlas of RNA Sequencing Profiles of Normal Human Tissues [43]. Total RNA isolation was performed using the RNeasy Mini Kit (Qiagen, Hilden, Germany, Cat. No. 74104), and RNA integrity number (RIN) was measured using an Agilent 2100 bioanalyzer (Agilent Technologies, Santa Clara, CA, USA). RNA concentration was measured using a Qubit RNA Assay Kit (Thermo Fisher Scientific, Cat. No. Q33222). Ribosomal RNA was depleted using the KAPA RNA Hyper Kit with RiboErase (Roche, Basel, Switzerland, Cat. No. 08098140702). Library concentrations and the length distribution of amplified cDNA were evaluated using the Qubit dsDNA HS Assay Kit (Life Technologies, Carlsbad, CA, USA, Cat. No. Q32851) and Agilent TapeStation (Agilent Technologies), respectively. Samples were sequenced using an Illumina NextSeq 550 (Illumina, San Diego, CA, USA) instrument to generate single-end reads with an average length of 75 bp and a targeted sequencing depth of approximately 30 million reads per sample. Primary quality control of the sequencing data was performed using the Illumina Sequencing Analysis Viewer (SAV) software (version 3.0). Demultiplexing was performed using the Illumina Bcl2fastq2 v2.17 (Illumina, San Diego, CA, USA) according to previously described methods [43]. For the RNA-seq pipeline, three independent biological replicates were performed for each condition (cell line × irradiation type × time point). Sequencing quality was assessed using FastQC (version 0.12.1); all samples exhibited Q30 scores > 90% and mapping rates to the GRCh38 reference genome via the STAR aligner exceeded 85%. All libraries were prepared and sequenced in a single batch.

### 4.7. Differential Expression and Bioinformatic Pathway Analysis

FASTQ files were aligned to the human reference genome (GRCh38) using STAR (v. 2.7.11b) in the GeneCounts mode guided by Ensembl GRCh38.89 transcriptome annotation [44]. Gene expression data were normalized and differential expression analysis was performed using the DESeq2 package [45]. Multiple-testing correction was implemented using the Benjamini–Hochberg false discovery rate (FDR) method. Genes exhibiting an adjusted *p*-value < 0.05 were considered significantly differentially expressed (DEGs).

Changes in the activation of intracellular molecular pathways of irradiated cells compared to unirradiated control cells were quantified using the Oncobox bioinformatics platform [46].

The pathway activation level (PAL), a computational metric that estimates the relative activity of predefined molecular pathways based on transcriptomic profiles, was calculated for each pathway using the following formula:

(10) $${PAL}_{p}=\sum_{n}{ARR}_{np}\times lnln\;{CNR}_{n}÷\sum_{n}{\left|{ARR}_{n}\right|}_{,}$$

where PAL_p_ is the level of activation of the molecular pathway p; CNR_n_ (case-to-normal ratio) is the ratio of the expression level of gene n in the irradiated sample to the average expression level of the unirradiated control group; *ln* is the natural logarithm; and the discrete ARR_np_ value (activator/repressor role) reflects the functional role of the gene *n* product within pathway p. Specifically, ARR_np_ is defined as −1 if the gene product *n* inhibits pathway p; 1 if *n* activates pathway p; 0 if *n* has an ambiguous or unclear role in a pathway p; and 0.5 or −0.5, if *n* is predominantly an activator or inhibitor of the pathway, respectively.

Molecular pathways were obtained from the Reactome, KEGG, BioCarta, NCI Pathway Interaction, and Oncobox Primary databases. For the DNA repair analysis, 38 DNA repair-related pathways were initially evaluated. To focus on biologically relevant differences, only pathways with a PAL variation greater than 10 between the maximum and minimum values across the analyzed conditions were retained for visualization, resulting in a filtered subset of 19 pathways. Cell-cycle-associated pathways were selected based on the presence of the terms “*G1*”, “*G2*”, “*S phase*”, or “*Cell Cycle*” in the pathway title, whereas cell-death pathways were selected using the terms “*Apoptosis*” or “*Cell Death*”. For visualization purposes, the ten pathways with the highest positive and negative PAL values are presented.

Statistical and mathematical analyses of the experimental data were conducted using the Statistica 8.0 software (StatSoft, Tulsa, OK, USA). The results are presented as the mean of three independent biological experiments ± standard error of the mean (SEM). Statistical significance between groups was evaluated using the Student’s *t*-test. The slope coefficients of the dose–response curves were compared using the *Z*-test.

## 5. Conclusions

This study demonstrates that ultrashort pulsed electron beams generated by the AREAL accelerator elicit distinct biological responses compared to conventional X-rays in human non-small cell lung cancer (NSCLC) cells. Compared with the X-ray reference, AREAL irradiation was more effective (RBE = 1.2) in reducing the clonogenic survival of p53-wild-type A549 cells and resulted in a greater residual persistence of γH2AX foci in both A549 and H1299 cells. Transcriptome analyses further revealed distinct, p53-related pathway-level signatures associated with DNA repair, cell-cycle regulation, and cell death. Collectively, these findings provide new insights into the molecular mechanisms underlying the cellular response of NSCLC to ultrashort pulsed electron beams and support continued investigation of this emerging irradiation modality for cancer radiotherapy.

## Figures and Tables

**Table 1:** Characterization of the AREAL laser-generated electron beam.

AREAL Beam Parameters		UV Laser Parameters	
Beam charge (pC)	30	Wavelength (nm)	258
Electron energy (MeV)	3.6	Pulse energy (µJ)	200
Pulse duration (fs)	450	Repetition rate (Hz)	1–50
Pulse repetition rate (Hz)	1–50	Energy stability	<1%
Beam spot size (mm)	15	Beam divergence (mrad)	<0.3
Norm. emittance (mm-mrad)	<0.5	Beam diameter (mm)	2.0
RMS energy spread	<1.15%	-	-
Online dose information	Faraday cup	-	-

## Data Availability

The original contributions presented in this study are included in the article/Supplementary Material. Further inquiries can be directed to the corresponding author.

## Supplementary Materials

The following supporting information can be downloaded at: https://www.mdpi.com/article/10.3390/ijms27167215/s1.
