# Supplementary material for: Laser-Generated Ultrashort Pulsed Electron Beams Induce p53-Related Alteration of DNA Repair and Cell Death Pathways in Non-Small Cell Lung Cancer Cells
Source: Int J Mol Sci. 2026 Aug 13;27(16):7215. doi: 10.3390/ijms27167215 (PMC13513337; doi:10.3390/ijms27167215)
Supplement: Supplementary file 1 [file ijms-27-07215-s001.zip › ijms-4395265-supplementary.pdf]

Table S1: PAL values for significantly modulated pathways in A549 and H1299 cells exposed to ultrashort pulsed electron beams or X-rays.

A549					
AREAL			X-rays		
Pathway	Adjusted p-value	PAL	Pathway	Adjusted p-value	PAL
resolvin_D_biosynthesis	0.0113	229	BRCA1_Pathway_G1_S_arrest	0.0449	43
methylglyoxal_degradation_VI	0.0084	195	NCI_Role_of_Calcineurin_dependent_NFAT_signaling_in_lymphocytes_Pathway_(apoptosis)	0.0345	31
NCI_a6b1_and_a6b4_Integrin_signaling_Pathway_(hemidesmosome_assembly)	0.0084	168	reactome_Recruitment_of_repair_and_signaling_proteins_to_double_stand_breaks_Main_Pathway	0.0302	27
lipoxin_biosynthesis	0.0113	152	biocarta_rac1_cell_motility_signaling_Pathway_(actin_filament_organization)	0.0305	25
aspirin_triggered_resolvin_E_biosynthesis	0.01	151	NCI_FOXA2_and_FOXA3_transcription_factor_networks_Main_Pathway	0.0478	23
aspirin_triggered_resolvin_D_biosynthesis	0.01	151	KEGG_Lysine_degradation_Main_Pathway	0.0499	22
aspirin_trigger_d_ lipoxin_biosynthesis	0.01	151	reactome_Fatty_Acyl_CoA_Biosynthesis_Main_Pathway	0.0071	20
anandamide_degradation	0.0181	107	biocarta_rho_cell_motility_signaling_Pathway_(actin_filament_polymerization)	0.0478	19
NCI_Class_II_PI3K_signaling_events_mediated_by_Akt_Pathway_(glucose_import)	0.0088	89	Mismatch_repair_effect	0.0231	18
NCI_Endogenous_TLR_signaling_Pathway_(regulation_of_interleukin_10_production)	0.0203	87	reactome_ATM_mediated_phosphorylation_of_repair_proteins_Main_Pathway	0.0251	16
thyroid_hormone_metabolism_I_via_deiodination	0.0192	−82	Glucocorticoid_Receptor_Signaling_Pathway_Cell_Cycle_Arrest	0.0145	−36
thyronamine_and_iodothyronamine_metabolism	0.0192	−82	reactome_Histamine_receptors_Main_Pathway	0.0061	−38
reactome_cell_division_Main_Pathway	0.0109	−84	reactome_TAK1_activates_NFkB_by_phosphorylation_and_activation_of_IKKs_complex_Main_Pathway	0.0251	−41

NCI_PLK1_signaling_events_Pathway_(chromosome_segregation)	0.0092	−95	lipoxin_biosynthesis	0.0246	−48
UMP_biosynthesis	0.0184	−104	NCI_Syndecan_4_mediated_signaling_events_Pathway_(cell_migration)	0.0251	−56
reactome_E2F_mediated_regulation_of_RNA_replication_Main_Pathway	0.0191	−105	resolvin_D_biosynthesis	0.0246	−72
reactome_G1_S_Specific_Transcription_Main_Pathway	0.0191	−105	NCI_GMCSF_mediated_signaling_events_Main_Pathway	0.0251	−74
Reactome_Unwinding_of_RNA_Main_Pathway	0.015	−106	NCI_IL2_signaling_events_mediated_by_STAT5_Main_Pathway	0.0251	−74
reactome_Switching_of_origins_to_a_post_replicative_state_Main_Pathway	0.0129	−112	NCI_IL2_signaling_events_mediated_by_STAT5_Pathway_(CD4-positive_CD25-positive_alpha_beta_regulatory_T_cell_lineage_commitment)	0.0251	−74
biocarta_cheapones_modulate_interferon_signaling_Pathway_(apoptosis)	0.0146	−135	NCI_IL2_signaling_events_mediated_by_STAT5_Pathway_(activation_induced_cell_death_of_T_cells)	0.0251	−74

H1299					
AREAL			X-rays		
Pathway	Adjusted p-value	PAL	Pathway	Adjusted p-value	PAL
NCI_Regulation_of_Androgen_receptor_activity_Pathway_(Pathway_positive_regulation_of_transcription_via_GATA2)	0.0157	106	KEGG_Mineral_absorption_Main_Pathway	0.0432	46
reactome_ATP_sensitive_Potassium_channels_Main_Pathway	0.0397	102	biocarta_sonic_hedgehog_receptor_ptc1_regulates_cell_cycle_Pathway_(cell_cycle_arrest)	0.0114	38
sulfite_oxidation	0.0018	101	D-imyo-inositol_3456-tetrakisphosphatase_biosynthesis	0.0159	38
NCI_Role_of_Calcineurin_dependent_NFAT_signaling_in_lymphocytes_Pathway_(positive_T_cell_selection)	0.0003	99	reactome_Assembly_of_the_RAD51_ssDNA_nucleoprotein_complex_Main_Pathway	0.0278	31
D-mannose_degradation	0.0023	93	biocarta_brca1_dependent_ub_ligase_activity_Main_Pathway	0.0369	31
NCI_LPA_receptor_mediated_events_Pathway_(positive_regulation_of_dendritic_cell_cytokine_production)	0.0177	82	NCI_Regulation_of_retinoblastoma_protein_Pathway_(proteasomal_ubiquitin_dependent_protein_catabolic_process)	0.015	29
NCI_Endogenous_TLR_signaling_Pathway_(regulation_of_interleukin_10_production)	0.0456	81	ATM_Pathway_S-phase_arrest	0.0138	27
NCI_Endogenous_TLR_signaling_Pathway_(regulation_of_granulocyte_colony_stimulating_factor_production)	0.0456	81	biocarta_role_of_brca1_brca2_and_atr_in_cancer_susceptibility_Pathway_(Pathway_protein_ubiquitination_of_ATM_BRCA1)	0.0159	27
NCI_FGF_signaling_Pathway_(negative_regulation_of_apoptosis)	0.0078	80	reactome_Presynaptic_phase_of_homologous_DNA_pairing_and_strand_exchange_Main_Pathway	0.0472	27
methylglyoxal_degradation_VI	0.039	80	biocarta_role_of_brca1_brca2_and_atr_in_cancer_susceptibility_Main_Pathway	0.0024	26
reactome_Mitochondrial_transcription_initiation_Main_Pathway	0.0005	−76	NCI_Integrins_in_angiogenesis_Pathway_(angiogenesis)	0.0206	−40
UDP-iNi-acetyl-D-glucosamine_biosynthesis_II	0.002	−78	ERK_Signaling_Pathway_Cell_Survival	0.0024	−41
STAT3_Pathway_G1_to_S_Cell_Cycle_Progression	0.0006	−79	reactome_Electric_Transmission_Across_Gap_Junctions_Main_Pathway	0.0278	−44

reactome_FGFR1b_ligand_binding_and_activation_Main_Pathway	0.0012	−82	NCI_Atypical_NF_kappaB_Pathway_(Pathway_degradation_of_NFKBIA)	0.0458	−45
NCI_Signaling_events_mediated_by_Stem_cell_factor_receptor_c_Kit_Pathway_(megakaryocyte_differentiation)	0.0228	−83	NCI_Ceramide_signaling_Pathway_(regulation_of_nitric_oxide_biosynthetic_process)	0.0203	−50
NCI_Signaling_events_mediated_by_Stem_cell_factor_receptor_c_Kit_Pathway_(response_to_radiation)	0.0188	−93	STAT3_Pathway_G1_to_S_Cell_Cycle_Progression	0.0076	−51
NCI_CD40_CD40_Signaling_Pathway_(proteasomal_ubiquitin_dependent_protein_catabolic_process)	0.0015	−98	ILK_Signaling_Pathway_Opsozination	0.0007	−53
NCI_PLK2_and_PLK4_events_Main_Pathway	0.0003	−98	NCI_Signaling_events_mediated_by_HDAC_Class_I_Pathway_(proteasomal_ubiquitin_dependent_protein_catabolic_process)	0.0453	−53
NCI_PLK2_and_PLK4_events_Pathway_(regulation_of_centriole_replication)	0.0003	−98	NCI_Notch_signaling_Pathway_(skeletal_muscle_tissue_development)	0.0066	−81
NCI_Signaling_events_mediated_by_Stem_cell_factor_receptor_c_Kit_Pathway_(cell_proliferation)	0.0102	−99	reactome_Regulation_of_Commissural_axon_pathfinding_by_Slit_and_Robo_Main_Pathway	0.0128	−98
